# Does metformin improve vascular heath in children with type 1 diabetes? Protocol for a one year, double blind, randomised, placebo controlled trial

**DOI:** 10.1186/1471-2431-13-108

**Published:** 2013-07-16

**Authors:** Jemma Anderson, Alexia S Peña, Thomas Sullivan, Roger Gent, Bronwen D’Arcy, Timothy Olds, Brian Coppin, Jennifer Couper

**Affiliations:** 1 School of Paediatrics and Reproductive Health, University of Adelaide, Adelaide, Australia; 2 Department of Paediatric Endocrinology, Women’s and Children’s Hospital, Adelaide, Australia; 3 Department of Public Health, University of Adelaide, Adelaide, Australia; 4 Department of Medical Imaging, Women’s and Children’s Hospital, Adelaide, Australia; 5 Department of Nutrition and Dietetics, Women’s and Children’s Hospital, Adelaide, Australia; 6 School of Health Sciences, University of South Australia, Adelaide, Australia; 7 Department of Paediatrics, Flinders Medical Centre, Adelaide, Australia

**Keywords:** Endothelial function, Metformin, Type 1 diabetes, Child

## Abstract

**Background:**

Cardiovascular disease is the leading cause of mortality in Type 1 diabetes (T1D). Vascular dysfunction is an early and critical event in the development of cardiovascular disease. Children with T1D have vascular dysfunction therefore early interventions to improve vascular health are essential to reduce cardiovascular mortality in T1D. Metformin is an insulin sensitising agent which is known to improve vascular health outcomes in type 2 diabetes (T2D) and other individuals with insulin resistance. It has been used safely in children and adolescents with T2D for over 10 years. This study aims to assess the effect of metformin on vascular health in children with T1D.

**Methods/Design:**

This study is a 12 month, double blind, randomised, placebo controlled trial to determine the effect of metformin on vascular health in children (age 8–18) with T1D. The sample size is 76 with 38 children in the metformin group and 38 children in the placebo group. Vascular health and biochemical markers will be measured at baseline, 3, 6 and 12 months. Vascular function will be measured using flow mediated dilatation and glyceryl trinitrate mediated dilatation of the brachial artery and vascular structure will be measured with carotid and aortic intima media thickness, using standardised protocols.

**Discussion:**

This study will be the first to investigate the effect of metformin on vascular health in children with T1D. It will provide important information on a potential intervention to improve cardiovascular morbidity and mortality in this population at high risk from cardiovascular disease.

**Trial registration:**

Australia New Zealand Clinical Trials Registry ACTRN12611000148976

## Background

### Vascular dysfunction and cardiovascular disease in Type 1 diabetes

XCardiovascular disease remains the leading cause of mortality in Type 1 Diabetes (T1D), despite significant developments in management over the past 20 years [1,2]. Children with T1D have evidence of cardiovascular abnormalities such as vascular endothelial and smooth muscle dysfunction and increased intima media thickness [3-5]. Vascular dysfunction precedes clinically evident vascular disease [6] and is potentially reversible. Although vascular complications are rarely seen during childhood, their pathogenesis begins soon after diagnosis and accelerates during puberty [7]. Vascular dysfunction in T1D is also accelerated by poor glycaemic control, overweight and obesity, genetic predisposition and insulin resistance. Early interventions to improve cardiovascular health in T1D are essential to reduce the burden of cardiovascular morbidity and mortality.

The doubling in incidence of T1D in childhood in Australia over the last 20 years parallels the overweight/obesity epidemic in Western childhood populations. In addition, age of diagnosis of T1D has decreased over the last 20 years and younger age of T1D onset is associated in some populations with higher Body Mass Index (BMI) at diagnosis [8,9]. The T1D population is susceptible to the population shift in BMI, with 65% of adults in a US cohort with T1D in the overweight or obese BMI range [10]. Higher BMI is associated with higher intima media thickness (IMT) blood pressure and dyslipidaemia [11].

The prevalence of overweight and obesity in children with T1D is higher than the normal population, at just over 38.5% in a Dutch cohort [12], 22.1% in a US cohort (compared with 16.1% in the normal population) [13] and 31% in our local South Australian cohort [14]. This is associated with other cardiovascular risk factors including hypertension, dyslipidaemia, and metabolic syndrome. Overweight and obesity may have additional clinical consequences with the associated higher insulin resistance contributing to vascular disease [12,15].

Adolescence is a critical time in determining risk of future vascular complications [16] and is a time when vascular dysfunction is potentially reversible. Optimisation of diabetes control should be the initial strategy to improve vascular health in children and adolescents with T1D. However, glycosylated haemoglobin (HbA1c) levels are higher than target levels recommended for prevention of complications [17,18]. This is despite the advances in insulin delivery with insulin analogues and continuous subcutaneous insulin infusion (CSII) [19], highlighting the need to identify additional vascular protective strategies to prevent cardiovascular disease at its inception. The aim of this study is to determine whether metformin improves vascular health in children with T1D.

### Assessment of vascular health

#### ***Vascular function***

The endothelium is a key regulator of vascular function [20] and endothelial dysfunction occurs early in the development of atherosclerosis [21]. Vascular dysfunction can be measured by Flow Mediated Dilatation (FMD) and Glyceryl Trinitrate mediated Dilatation (GTN). These are early, non-invasive markers of atherosclerosis and predate the development of clinically evident disease [22,23].

Ultrasound assesses brachial artery responses to increased blood flow (FMD). This induces nitric oxide release from the endothelium with a resultant increase in brachial artery diameter and is a measure of endothelial function. Exogenous administration of glyceryl trinitrate increases vessel diameter independent of the endothelium and is a measure of smooth muscle function (GTN). FMD and GTN have proven to be accurate and reproducible methods for assessment of vascular function [24].

Endothelial dysfunction of the coronary arteries, as measured by coronary artery vasomotor responses, is associated with a higher incidence of cardiovascular events in adulthood. FMD of the brachial artery relates to both coronary artery vasomotor responses and to the extent of coronary artery disease on coronary angiography findings [25]. Reduced FMD in adults relates to traditional cardiovascular risk factors [22]. FMD is abnormal in children and young adults at risk of atherosclerosis [23]. FMD and GTN will be outcome measures in this study.

#### ***Vascular structure***

The thickness of the intima and media layer of the carotid (cIMT) and aortic (aIMT) walls are structural markers of atherosclerosis and can be evaluated by ultrasound.

cIMT is a well-established index of early atherosclerosis that correlates with prevalent and incident coronary heart disease and stroke [26]. Relative risk of stroke or myocardial infarction in older adults without a history of cardiovascular disease increases by 35% with an increase in cIMT of 1 SD, i.e. a relatively large clinical effect for a small change in cIMT [26]. Overweight and obese children have increased cIMT compared with age matched controls [27]. cIMT has been used as a primary outcome measure in landmark intervention trials [7,28]. Diet and exercise improve endothelial dysfunction and reduce cIMT in obese children over 12 months [29]. In this study FMD improved early and was maintained and cIMT improved at 12 months. Atherosclerosis begins in the abdominal aorta. We and others have shown that aIMT precedes changes in cIMT in children with accelerated atherosclerosis, including T1D [3,4]. cIMT and aIMT will be used in this study as structural outcome measures.

#### ***Adiponectin***

Adiponectin is an adipocytokine that regulates nitrous oxide by activating endothelial nitric oxide synthase. It may provide a measurable link between visceral obesity, insulin resistance and vascular dysfunction. We have shown that adiponectin levels relate to vascular smooth muscle function in obese youth [30]. Adiponectin/leptin ratio is an emerging measure of insulin resistance and is substantially higher in children with type 2 compared with type 1 diabetes [31,32].

### Metformin as a therapy to improve vascular health

#### ***Mechanism of action***

Metformin is a biguanide that reduces glucose output from the liver and increases insulin sensitivity. Metformin activates the energy regulating AMP activated protein kinase (AMPK), principally in muscle and liver. This is the major mechanism of metformin’s action, to increase insulin stimulated glucose uptake in skeletal muscle and adipocytes, and reduce hepatic glucose output. Metformin also activates AMPK in the endothelium and smooth muscle. This is likely to explain the improvement in endothelium dependent and independent vascular responses with the administration of metformin in adults with T1D and polycystic ovarian syndrome [33-35]. It may also explain the recognised benefits of metformin on cardiovascular risk independent of its glucose lowering effect. Metformin also improves adiponectin and leptin levels [35-37].

#### ***Type 2 diabetes***

Metformin is the first line medication (with diet and exercise) in youth with type 2 diabetes (T2D) and/or with metabolic syndrome [38]. In T2D it improves endothelial function, decreases weight gain, triglycerides, LDL cholesterol and pro-inflammatory and pro-coagulation factors [39,40]. Importantly, in two large randomised trials metformin reduced the rate of myocardial infarction in adults with T2D by 33%. Metformin also improves BMI, body composition and fasting insulin in obese youth without diabetes [41,42].

#### ***Type 1 diabetes***

A 6 month pilot study reported improvements in vascular health, as measured by FMD, in 44 adults with T1D treated with metformin or placebo in addition to insulin [33]. Meta-analysis of randomised studies of metformin in T1D shows benefits in reducing daily insulin requirement and reduced weight gain [43]. The potential benefit on improving LDL cholesterol is not confirmed and reductions in HbA1c are inconsistent.

There are only two studies in children using metformin in addition to insulin. They show that metformin lowered HbA1c and decreased insulin dose without weight gain in children with poor metabolic control [44,45].

Therefore, metformin is a logical adjunct treatment for T1D in addition to insulin. There are no data on the effects of metformin on vascular health in children and adolescents with T1D. We have shown that children with T1D have severe vascular dysfunction which relates to BMI [14]. We will therefore determine the effect of metformin on vascular function and structure in children with T1D and above average BMI, in the absence of any published trials.

### Aims and hypotheses

The *primary objective* of this double blind, randomised, placebo controlled trial is to determine whether metformin improves vascular function as measured by FMD in children aged 8–18 years who have T1D.

The *secondary objectives* are to determine the effect of metformin on vascular health as measured by GTN, cIMT and aIMT in addition to other variables including adiponectin/leptin ratio, waist circumference, BMI, body composition, insulin requirements, HbA1c and lipids.

We hypothesise that the intervention group (those that receive metformin) will have a significant improvement in vascular health compared to the placebo group. We also hypothesise that those in the intervention group will have improvements in adiponectin/leptin ratio, waist circumference, BMI, body composition, insulin requirements, HbA1c and lipids.

## Methods/design

### Approval and registration

This study is a parallel, double blind, randomised placebo controlled trial over 12 months in two paediatric diabetes centres in Adelaide, South Australia. The trial has been approved by the Women’s and Children’s Hospital Research Ethics Committee (HREC 2327/12/13) and Flinders Medical Centre Research Ethics Committee (HREC 443.12) and is prospectively registered with the Australian New Zealand Clinical Trials Registry (ACTRN12611000148976).

All children with T1D who are seen in the diabetes outpatient clinics of the Women’s and Children’s Hospital (WCH) and Flinders Medical Centre (FMC) paediatrics department will be approached consecutively to be screened for eligibility in the study.

A total sample of 76 children aged 8–18 years with T1D will be recruited. The treatment period is 12 months in duration. Written informed consent will be obtained from all parents of participants and written assent will be obtained from all participants.

### Inclusion criteria

Each child must meet the following criteria to be involved in this study:

• diagnosed with type 1 diabetes

• aged between 8 and 18 years.

• BMI > 50th centile for age and sex [Centers for Disease Control and Prevention 2000 standardized reference charts (http://wwwn.cdc.gov/epiinfo/)].

• T1D duration greater than 1 year

• insulin requirements > 0.5 units /kg/day to exclude subjects in the remission phase of T1D

### Exclusion criteria

Children are excluded from the study if they meet any of the following criteria:

• non T1D i.e. T2D or other forms of diabetes

• severe hypoglycaemic episode in preceding 6 months defined as a loss of consciousness or convulsion associated with hypoglycaemia

• recurrent diabetic ketoacidosis (more than 2 episodes in the preceding year)

• other serious co-morbidities but not including treated hypothyroidism or coeliac disease

• contraindications to metformin therapy: hypersensitivity to metformin, renal or liver dysfunction, vitamin B12 deficiency, inability to abstain from alcohol

• pregnancy or breast feeding

• subjects taking metformin, statins, multivitamins, or anti-hypertensives

### Randomisation

The study has a planned sample size of 76 with 38 in each group. Randomisation was performed by an independent statistician using statistical software S-plus version 8.1 to provide equal representation of placebo and metformin. Allocation concealment was used to implement the random sequence allocation.

Participants will be recruited from paediatric outpatient clinics at WCH and FMC by a single investigator (JA). Participants will be assigned randomisation numbers by the investigator in sequence and allocated to a treatment group by the pharmacist using the generated randomisation list. Treatment allocation will be concealed from all study investigators, the distributing pharmacist and participants to minimise potential bias.

Analysis of the data will be performed by a statistician (TS) who was not involved in the initial randomisation.

### Participant withdrawal

Any participant may terminate participation in the study without giving a reason and without any disadvantage or interference with the diabetes care provided to the participant at the treating hospital. An investigator can stop the participation of a subject after consideration of the benefit/risk ratio of participation in the trial. Reasons to stop participation include: serious adverse events, request by safety committee of early termination due to safety reasons and limitation to continue the trial such as a participant moving interstate.

### Treatment arms and dosage of medication

**Figure 1:**
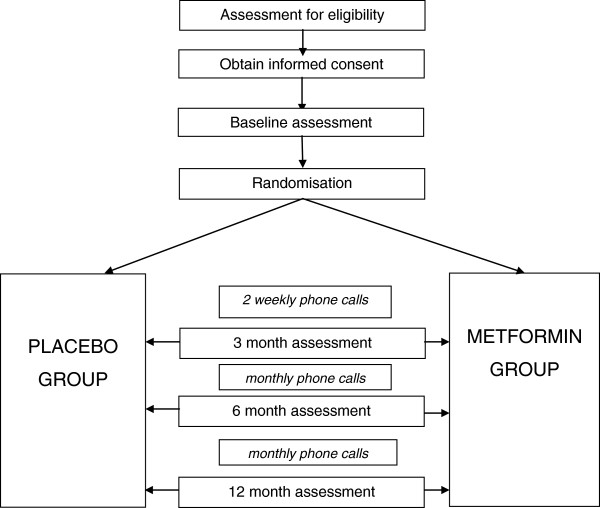
Study procedure details.

The children will receive either metformin or placebo according to their randomly allocated group. The study procedure details are outlined in Figure 1. Metformin 500 mg oral tablets will be supplied by GenericHealth (Camberwell, Victoria, Australia). The placebo will be also supplied by the same company and is identical in appearance and ingredients, aside from the active ingredient, metformin hydrochloride. If the participant’s weight is less than 60 kg they will be assigned to a dosage of 500 mg twice a day (titrated up over 2 weeks). If the participant weighs greater than 60 kg they will be assigned to a dosage of 1 g twice a day (titrated up over 6 weeks). Dose titration will be limited to tolerance of the medication. If a participant experiences a reduction in insulin dose of greater than 20%, or if they have persistent gastrointestinal side effects, they will be prescribed the maximum tolerated dose.

Both groups will also receive a dietary intervention at the commencement of the study and at 3 months. At the first visit, all subjects will receive standardised information regarding healthy diet, portion sizes, regular meals and physical activity. This information will be delivered using a ‘Type 1 diabetes and healthy weight’ resource Additional file 1), developed by our dietician (BD) using current guidelines for healthy eating in T1D.

At the 3 month assessment, all participants will take part in a standardised interactive nutrition session developed by BD comparing the relative nutritional value of healthy snack foods to ‘sometimes’ and ‘treat’ snack foods.

### Outcome measures

Table 1 shows which primary and secondary outcomes will be measured at different time points in the study. Table 2 shows additional information which will be collected at the assessments and Table 3 shows the additional information collected at each telephone call (which occurs 2 weekly for the first 3 months and monthly thereafter).

**Table 1:** Primary and secondary outcome measures

	**Baseline**	**3 months**	**6 months**	**12 months**
**Primary outcome**				
Vascular endothelial function (FMD)	*	*	*	*
**Secondary outcomes**				
Vascular smooth muscle function (GTN)	*	*	*	*
Vascular structure (cIMT and aIMT)	*			*
Physical examination	*	*	*	*
- anthropometric measurements (height, weight, BMI, waist circumference)				
- the presence of acanthosis nigricans				
- severity of acne				
- ferriman - gallwey score (girls)				
Frequency and severity of hypoglycaemia	*	*	*	*
Insulin dose (units/kg/day)	*	*	*	*
Glycosylated haemoglobin	*	*	*	*
Fasting glucose	*	*	*	*
Fasting total/HDL/LDL cholesterol/triglycerides	*	*	*	*
High sensitivity C-Reactive Protein (HsCRP)	*	*	*	*
Early morning urinary albumin/creatinine	*	*	*	*
Urinary prostaglandin F2α	*	*	*	*
Liver function, renal function tests and lactate	*	*	*	*
Total Plasma homocysteine	*	*	*	*
Girls only: Testosterone, free androgen index, Sex Hormone-Binding Globulin, Dehydroepiandrosterone, Anti-Mullerian Hormone, 17-hydroxy progesterone, prolactin, Luteinizing hormone, Follicle Stimulating Hormone, progesterone & oestradiol	*	*	*	*
Total Adiponectin	*	*	*	*
Leptin	*	*	*	*
Body composition (bio-electrical impedance analysis using Tanita body composition scales)	*	*	*	*
Retinal photograph	*			*
Total body Dual-energy X-ray absorptiometry scan	*			*

*Indicates assessment of a variable at the timepoint indicated.

**Table 2:** Additional information collected at assessments

	**Baseline**	**3 months**	**6 months**	**12 months**
ACAES questionnaire v1.2	*	*	*	*
Physical examination – tanner stage	*	*	*	*
Daily energy expenditure (SenseWear arm band)	*	*	*	*
Family history of premature cardiovascular disease	*			*
Pregnancy test in post menarchal girls	*	*	*	*
Serum cotinine	*	*	*	*
Adherence data using MEMS caps and manual tablet count	*	*	*	*
Serum Vitamin B12, Folate, red cell folate	*	*	*	*

* Indicates assessment of a variable at the timepoint indicated.

**Table 3:** Additional information collected with each phone call

	**0-3 months**	**3-12 months**
Side effects	2 weekly telephone calls	Monthly telephone calls
- hypoglycaemia (number, severity)		
- gastrointestinal symptoms (nausea, vomiting, diarrhoea, anorexia)		
- rash		
- other side effects		
Insulin requirements		

### Ultrasound assessment of vascular function (FMD and GTN) and vascular structure (cIMT and aIMT)

Experienced and blinded sonographers (trained and led by RG) will perform all B mode ultrasound examinations with a 17–5 MHz linear array transducer (iU22; Phillips, Bothel, Washington, USA) in a temperature-controlled room (22–24°C).

#### ***Flow mediated dilatation (FMD) and glyceryl trinitrate mediated dilatation (GTN)***

FMD and GTN will be assessed as previously described [3,14,46,47]. Ultrasound images of the brachial artery in longitudinal section, 2–15 cm above the elbow will allow measurements of the arterial diameter. Each study will include 4 scans: (1) resting scan; subsequently, reactive hyperaemia is induced by occluding arterial blood flow using a sphygmomanometer inflated to 250 mmHg for 4 minutes; (2) FMD scan recorded between 45–75 seconds after cuff deflation; (3) re-control scan 10–15 minutes later (allowing for vessel recovery); and (4) last scan, taken 4 minutes after the sublingual administration of the GTN spray (400 μg, Nitrolingual Pump spray, manufactured by G. Pohl-Boskamp GmbH & co. KG Hohenlockstedt, Germany, distributed by Sanofi-Aventis, Macquarie Park, NSW, Australia). For each scan, measurements will be made over 4 consecutive cardiac cycles, incident with the R wave on the ECG (i.e., at the end of diastole), by observers blinded to the intervention type, using ultrasonic calipers. Measurements will be averaged and expressed as percentages of the resting scan. Our coefficient of variation (CV) between 20 controls is 3.9% for FMD and 4.0% for GTN-mediated dilatation [46].

#### ***Carotid and aortic intima media thickness (cIMT and aIMT)***

cIMT and aIMT will be assessed as previously described [3,48]. In brief, the left and right common carotid artery will be imaged in a standardized magnification (2 × 2 cm) using images of the posterior (far) wall of the distal 10 mm of the common carotid artery, just proximal to the carotid bulb. Optimal images will be captured at the end of diastole, incident with the R-wave of the ECG, for later analysis. The greatest distance between the lumen-intima interface and media –adventitia interface (IMT) is measured using an automatic edge detection and measurement computer software package, and mean and maximum IMT are recorded. The mean of 2 measurements for aIMT and 6 measurements (3 on each side) for cIMT will be calculated according to the current Gold Standard protocol for cIMT. Analysis will be by two blinded observers. Sonographers have been trained and have undergone an accreditation process to evaluate the quality of their scans for this study in 2009. Our inter-observer intra-class coefficient for cIMT measurements is 0.99 (CV 1.2%) and the intra-observer intra-class coefficient for cIMT measurements is 0.97 (CV 2.4%) [3].

#### ***Physical examination***

Weight and body composition will be measured in light clothing using BC-418 segmental body composition analyser (Tanita, Tokyo, Japan distributed by Wedderburn, Inglewood, NSW, Australia). Height will be measured on a wall mounted stadiometer (to 0.1 cm). Waist and hip circumference will be measured with a tape (to 0.5 cm). Waist circumference will be measured at the midpoint between the lower edge of the ribs in the mid-axillary line and the top of the iliac crest, at minimal respiration. Hip circumference will be measured at the maximum circumference of the buttocks with the tape parallel to the floor. BMI z score will be calculated using 3.2 EpiInfo database version.2 and Centers for Disease Control and Prevention 2000 standardized reference charts (http://wwwn.cdc.gov/epiinfo/). Blood pressure will be measured using DINAMAP (Carescape V100 Vital signs monitor, GE Healthcare, Milwauke, WI) with appropriate-size cuff on the left arm after 10 minutes of rest in supine position. The mean of 3 consecutive measurements will be recorded.

#### ***Laboratory methods***

Cholesterol (LDL and HDL) and triglycerides will be measured using commercial enzymatic assays on Roche/Hitachi cobas C systems. High sensitivity C reactive protein (hs CRP) will be measured using a near infrared particle immunoassay method using IMMAGE Immunochemistry Systems Reagent (Beckman Coulter Inc, Fullerton, California, USA). Glycosylated haemoglobin (HbA1c) will be measured using a latex immunoagglutination inhibition methodology (DCA 2000 Hemoglobin A1c Reagent Kit; Bayer, Toronto, Ontario). Serum cotinine will be measured using a cotinine micro-plate EIA (STC Technologies). Urinary albumin/creatinine will be measured by immunoturbidometric and enzymatic colorimetric methods in routine laboratory assays. Oxidative stress will be assessed by measuring urinary PGF2α using a competitive enzyme-linked immunoassay and values obtained will be corrected for urinary creatinine. Liver function and renal function tests will be measured by Roche/Hitachi cobas C systems.

Serum samples will be frozen at -80 degrees Celsius for later measurement of adiponectin, leptin and androgens. Total adiponectin and leptin will be analysed using an enzyme-linked immunoassay (ALPCO diagnostics, Salem, NH, USA). Serum androgens will be measured by liquid chromatography mass spectroscopy (CPR Pharma Services, Adelaide, South Australia).

#### ***Total body dual-energy X-ray absorptiometry scan***

The ratio of total body fat (g) to lean body mass (g) will be measured using dual-energy X-ray absorptiometry (DXA) on a GE-Lunar Prodigy machine (GE LunarCorp, Maddison, WI) equipped with adult property software, version 3.6, which is appropriate for body weight of subjects 8–18 years. Fast scan mode and standard subject positioning will be used for these measurements. Mean precision of our machine is 1.3% for soft tissue.

#### ***Assessment of energy intake and diet quality***

The Australian Child and Adolescent Eating Survey (ACAES – version 1.2) [49] is a food frequency questionnaire based on the previous 3–6 months of food intake across all food categories. It will be interviewer-administered by the same dietitian at each assessment. At baseline, the ACAES will calculate the dietary intake over the previous 6 months. At subsequent assessments (3, 6 and 12 months) the ACAES questionnaire will calculate the dietary intake since the previous assessment. The ACAES will determine total caloric intake and macro and micronutrient content of the participant’s diet.

### Assessment of energy expenditure

Daily energy expenditure will be measured at 0, 3, 6 and 12 months. This will be assessed with the use of a SenseWear MF (Mini Form factor) device (analysed with SenseWear Software Version 7.0. Temple Healthcare Pty Ltd, Bowral, Australia) which is an external armband worn in this study for 5 days before each assessment time point. Based on measurements of accelerometry, heat flux, galvanic skin response, skin temperature, near-body temperature and patient characteristics (gender, age, height, weight) proprietary algorithms will calculate energy expenditure, number of steps taken and sleep duration.

### Monitoring of safety and adverse events

#### ***Blood glucose management during study***

All subjects will receive 2 weekly telephone review of blood glucose control for the first 3 months and monthly thereafter. They will also have access to a 24 hour phone hotline serviced by one of 4 paediatric endocrinologists for acute insulin dose adjustment.

#### ***Compliance assessment***

Assessment of adherence to the study medication regimen will be assessed at each assessment time point (3 months, 6 months and 12 months). All unused study drugs will be returned to the investigator for counting. MEMS caps (AARDEX group LTD, Sion, Switzerland) will also be utilised to measure compliance. These caps record how many times the study medication bottle is opened between study visits and the data will be downloaded for review and analysis. Compliance will be encouraged at the 3 month and 6 month study visit using the downloaded data from the MEMS cap.

### Monitoring of adverse events

Renal, liver function tests and lactate will be performed at 0, 3, 6 and 12 months. Metformin can decrease levels of vitamin B12, so children with vitamin B12 deficiency will be excluded and vitamin B12 will be monitored during the study. The risk of hypoglycaemia will be monitored and insulin will be adjusted accordingly.

Metformin can cause nausea and diarrhoea. Any illness or need for additional medication in a subject will be recorded throughout the study. Letters to the subjects’ endocrinologists and general practitioner advising of entry into the trial will be sent, and the WCH and FMC emergency departments will have a record of the subject’s participation in the trial in the medical records.

All adverse events will be reported to the medication safety committee for the trial in a monthly monitoring update. All serious adverse events will be reported to the medication safety committee and the WCH human research ethics committee within 24 hours of the event occurring. Serious adverse events occuring in FMC participants will also be reported to the FMC human research ethics committee. A serious adverse event is defined as one which is fatal or life threatening or requires hospitalization.

### Data analysis

All analyses will be performed under the intention to treat principle. The primary outcome, FMD, will be compared between metformin and placebo groups over 12 months using a linear mixed effects model. Linear mixed effects models will also be used to compare changes in secondary continuous outcomes (GTN, adiponectin/leptin ratio, lipids, HbA1c, insulin requirements, cIMT and aIMT) between treatment groups (metformin/placebo) over the four reviews. The linear mixed effects models will account for the within-subject correlations that are produced when repeating outcome measurements in individual subjects over time. Both unadjusted and adjusted analyses will be performed, with adjustment for important pre-specified baseline covariates including age, sex and BMI.

While no studies have looked at the effect of metformin on FMD in T1D in this age group, our previous work found an improvement in FMD of 3.1% with an SD of 4.3 in children with T1D receiving folic acid over 8 weeks [46,47]. Assuming an equivalent improvement with metformin from baseline to 12 months, we would require 32 subjects per group to have 80% power to detect a difference in FMD changes of 3.1% between randomised groups (alpha = 0.05 two-sided). Our dropout rate in previous studies in this population with similar intervention and investigation over 24 weeks has been 1–2%. We have conservatively factored in a dropout rate of 15% giving a total recruitment required of 76 subjects (38 in each group).

## Discussion

Cardiovascular disease is the commonest cause of morbidity and mortality in T1D. The origins of cardiovascular disease are in childhood. Good metabolic control is essential in the prevention of cardiovascular disease however targets for optimal glycaemic control are often not met in children and adolescents with T1D. It is crucial to investigate additional effective and early interventions to prevent or reduce vascular pathology in this population. Metformin is a good candidate for early prevention of cardiovascular disease as it has been prescribed in youth with T2D for over 10 years and reduces cardiovascular disease in T2D.

Metformin is generally well tolerated and easy to administer with a good safety profile. Vascular health outcomes will be measured using non invasive, valid and reliable measurements by experienced investigators. This is, to our knowledge, the first study to examine whether administration of metformin will improve vascular health in children with T1D. Determining the effect of metformin on vascular health in children with T1D will provide a potential early intervention for cardiovascular disease in this population.

## Abbreviations

ACAES: Australian child and adolescent eating survey; aIMT: Aortic intima media thickness; AMPK: AMP activated protein kinase; BMI: Body mass index; cIMT: Carotid intima media thickness; CSII: Continuous subcutaneous insulin infusion; DXA: Dual-energy X-ray absorpitometry; FMC: Flinders Medical Centre; FMD: Flow mediated dilatation; GTN: GTN mediated dilatation; HbA1c: Glycosylated haemoglobin; IMT: Intima media thickness; T1D: Type 1 diabetes; T2D: Type 2 diabetes; WCH: Women’s and Children’s Hospital.

## Competing interests

The authors declare that they have no competing interests.

## Authors’ contributions

JC and AP received study funding. AP, JC and JA conceived of the study. All authors participated in study design. TS performed sample size calculation, planned the statistical analysis and will be involved in study analysis. RG, lead sonographer, reviewed the vascular health assessment methods and will supervise scan quality. BD designed the dietary intervention and created the resource “Type 1 Diabetes and Healthy Weight”. TO assisted in evaluation of physical activity and energy expenditure for the study. BC assisted with the ethics application for FMC and will supervise the FMC patients. JA, AP and JC drafted the manuscript. All authors read and corrected draft versions and approved the final manuscript.

## Pre-publication history

The pre-publication history for this paper can be accessed here:

http://www.biomedcentral.com/1471-2431/13/108/prepub

## Supplementary Material

Additional file 1 “Type 1 Diabetes and healthy weight” dietician resource used for dietary intervention at baseline assessment. Click here for file
