# Supplementary material for: Does metformin improve vascular heath in children with type 1 diabetes? Protocol for a one year, double blind, randomised, placebo controlled trial
Source: BMC Pediatr. 2013 Jul 16;13:108. doi: 10.1186/1471-2431-13-108 (PMC3728146; doi:10.1186/1471-2431-13-108)
Supplement: Additional file 1 — “Type 1 Diabetes and healthy weight” dietician resource used for dietary intervention at baseline assessment. [file 1471-2431-13-108-S1.pdf]

Type 1 Diabetes and Healthy Weight

Having a healthy weight is vital for everyone but especially when living with type 1 diabetes. Excess weight can affect insulin action and overall health. Healthy eating, appropriate insulin adjustments and active lifestyle are the best ways to achieve and maintain healthy weight.

Here are some quick tips to ensure you are eating well and being active.

1. Eat three main meals every day: breakfast, lunch and dinner.
2. Drink water
3. Eat more low GI carbohydrates
4. Eat healthy snacks in between meals, but only if you need it
5. Eat less fat
6. Think before you eat more
7. Enjoy meals with your family with the television (TV) switched off
8. Cut down on screen time
9. Be active for at least 60 minutes a day

Make healthy food choices

1. Eat three main meals every day: breakfast, lunch and dinner

Whether you are on insulin injections or an insulin pump, it is important to have three main meals per day. Skipping meals, especially breakfast, often leads to over eating at the next meal or snack.

When planning your meals make sure you:

> Cut down on portion size.

> Choose a wide variety of foods from each food group:

– two serves of fruit and five serves of vegetables each day

– high fibre carbohydrates such as wholemeal/wholegrain breads, cereals, pasta and rice

– smaller amounts of low fat dairy foods, lean meats, poultry, fish, eggs, nuts, and fats and oils.

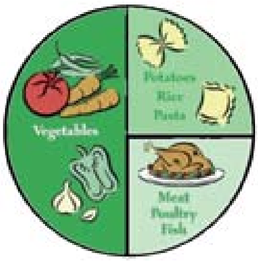

Try and make your plate look like the example to the right

2. Drink water

> Water should be the main drink for everyone.

> Avoid high sugar drinks such as regular soft drink, regular cordial and sports drinks as they will affect your blood glucose level.

> Limit unsweetened juice and reduced fat flavoured milks to only one glass per day.

3. Eat more low GI carbohydrates

These will help you feel full for longer and help to control blood glucose levels. It's easy to switch to the low GI – healthy options:

High GI	Change to	Lower GI (Healthy Option)
White bread	→	Wholegrain bread
Corn flakes of rice bubbles	→	Special K or porridge
Jasmine rice	→	Basmati, brown or Doongara rice
White potato	→	Sweet potato
Cruskit	→	Vita Weats

Women's & Children's Hospital

4. Eat healthy snacks in between meals, but only if you need it

Some insulin regimens do not require you to eat snacks in between meals (pumps or basal bolus regimes). If you need to eat snacks between meals, switch from high sugar/high fat options to healthier options.

Go for these healthy snacks		Snacks to limit/avoid
Worth 1 exchange	*Free or low carbohydrate options*	
One piece of fruit	Carrot and cucumber sticks with salsa or low fat cream cheese/dip	Chips/crisps
Small tub of diced fruit in natural juice	~ Half a cup of strawberries or other berries	Muesli bars, cereal bars, breakfast bars or fruit bars
Small tub of low fat yoghurt	Veggie Skewers – skewer cucumber, cherry tomatoes and low fat cheese onto a kebab skewer stick and dip into salsa, a tsp of mashed avocado or low fat cream cheese	Chocolate bars or lollies
Three wholegrain crackers (ie Vita-Weats) with Vegemite or low fat cheese and tomato	Small bowl of salad topped with low fat grated cheese and sweet chilli sauce	Fruit juice – avoid fruit drink and limit unsweetened juice to one glass per day
Three cups of homemade plain popcorn	Small bowl of homemade vegetable soup (including vegetables such as pumpkin, cauliflower, carrot, leek, tomato etc, and with no potato)	Ice blocks or ice creams
Small packet of pretzels	A handful (10–15) of raw unsalted almonds, cashews or walnuts (Maximum 3–4 times per week)	Muffins, cakes or buns
¼ –½ cup of wholegrain breakfast cereal with low fat milk	A small boiled egg (sliced) with tomato, lettuce and other vegetables (Maximum 2–3 times per week)	Savoury biscuits (eg shapes, Jatz etc.)
One crumpet, one piece of toast or two wholemeal pikelets topped with a spread of Vegemite.		Sweet biscuits (eg Tim Tams, Oreos, tiny teddies etc.)
Three homemade vegetable cold rolls (no noodles)		Pastries and bakery items (eg sausage rolls, custard tarts etc.)

5. Eat less fat

- Use low fat or reduced fat foods.
- Limit margarine, butter, oil, cream and mayonnaise.
- Use lean meats/chicken and remove visible fat or skin.
- Choose foods that have been grilled or steamed and avoid food which is battered, crumbed or fried.
- Avoid high fat snacks like chips, chocolate, biscuits, pastries and cake.
- Avoid takeaway foods.

6. Think before you eat more

Wait 20 minutes after you have finished eating to see if you are still hungry. If you are, head for the salad or vegetables (other than potato).

7. Enjoy meals with your family with the TV switched off

Distractions such as TV can cause you to overeat.

8. Cut down on screen time

Less than two hours of screen time a day is best. Screen time includes watching TV, being on facebook, time at the computer and playing video games.

9. Be active for at least 60 minutes a day

This can be all at once or in two–three blocks of 20–30 minutes.

- Play a team sport such as basketball, netball, soccer, football or cricket.
- Have a go at swimming, dancing, martial arts, bike riding. roller blading, skateboarding, climbing, ball games, or jump on the trampoline.
- Be active wherever you can.

Take the stairs instead of the lift, walk or ride your bike to school, or get off the bus a few stops early and walk the rest of the way.

Produced by

Children, Youth and Women's Health Service
Nutrition and Endocrinology/ Diabetes Departments
72 King William Road
North Adelaide SA 5006
Phone (08) 8161 7233

BY NC ND
http://www.gilf.gov.au/

Government of South Australia
SA Health

Women's & Children's Hospital

© Department of Health. All rights reserved.
Printed April 2011. Designed by Digital Media (4305S).
